# Problematic social networking use and loneliness among college students: a serial mediation model of basic psychological need satisfaction and self-acceptance

**DOI:** 10.3389/fpsyg.2026.1944813

**Published:** 2026-09-11

**Authors:** Xiuju Tian, Lijun Wang

**Affiliations:** 1 College of Psychology, Zhejiang Normal University, Jinhua, Zhejiang, China; 2 Mental Health Education Center, Huzhou Normal University, Huzhou, Zhejiang, China

**Keywords:** problematic social network use, loneliness, basic psychological need satisfaction, self-acceptance, serial mediation

## Abstract

**Introduction:**

This study investigates the association between problematic social network use and loneliness among university students, framed by Self-Determination Theory.

**Methods:**

A sample of 992 undergraduates (mean age = 20.31 years; 488 males, 49.2%; 504 females, 50.8%) from three universities in Zhejiang, a province in Southeast China, completed standardized measures. Serial mediation analysis using PROCESS Model 6 examined whether basic psychological need satisfaction and self-acceptance sequentially mediate this relationship.

**Results:**

Results confirmed a positive direct link between problematic social network use and loneliness. Significant indirect effects emerged through need satisfaction (β = 0.133, 95% *CI* [0.104, 0.165]), self-acceptance (β = 0.045, 95% *CI* [0.024, 0.067]), and their serial pathway (β = 0.061, 95% *CI* [0.046, 0.078]), accounting for 26.28%, 8.89%, and 12.06% of the total association, respectively. All bootstrap confidence intervals excluded zero.

**Conclusion:**

Problematic social network use appeared alongside diminished psychological need fulfillment, lower self-acceptance, and elevated loneliness. This pattern of co-occurrence describes how digital behaviors and mental health states are distributed across participants. Future research should test these pathways longitudinally and across diverse cultures. Practically, interventions targeting need satisfaction and self-acceptance rather than merely reducing screen time could more effectively mitigate loneliness in higher education settings.

## 1 Introduction

Loneliness (LON) refers to the distressing emotional state that arises when individuals perceive a qualitative or quantitative deficiency in their social relationships (Fan et al., 2024). Within Erikson’s theory of psychosocial growth, the period of higher education constitutes a formative window when students work through the opposing poles of— intimacy attainment and social withdrawal (Munley, 1977). Failure to successfully navigate this stage may have enduring consequences for psychological wellbeing. Indeed, LON has emerged as a pervasive concern among college students worldwide. Cross-national surveys indicate that 93.3% of Australian college students (Xiong et al., 2025) and 78.98% of their UK counterparts (Akram et al., 2025) reported moderate to high levels of LON. Among Chinese college students, a prevalence rate of 55.2% was documented during the COVID-19 pandemic (Li et al., 2025), and this figure is particularly concerning given China’s large university population. Moreover, LON in college students has been robustly linked to a range of adverse outcomes, including depression, anxiety, suicidal ideation, and diminished academic performance (Bruss et al., 2024; Li et al., 2021). Despite its high prevalence and significant ramifications, the psychological mechanisms underlying LON in this population remain insufficiently understood.

One source of individual differences in LON is problematic social network use (PSNU), characterized by compulsive, over engaged, and dysregulated social media behavior that hampers normal functioning (Shannon et al., 2025). Both external behavioral factors, such as excessive social media engagement, and contextual factors, such as reduced opportunities for face-to-face interaction, have been consistently associated with higher levels of LON (González-Nuevo et al., 2023; Jiang, 2021; Xu et al., 2023). In addition to these external influences, internal psychological processes may also play a critical role. In particular, the frustration of basic psychological needs and low self-acceptance have been identified as important psychological vulnerabilities associated with LON (Bruss et al., 2024; Li et al., 2021). Psychological need satisfaction (PNS) denotes the degree to which one’s needs for autonomy, competence, and relatedness are met (Liu et al., 2013). Within the PSNU context, unfulfilled psychological needs may correspond with perceived deprivation and inner tension, and these experiences show links to higher LON (Cheng and Lau, 2022). Meanwhile, self-acceptance (SA) is a positive psychological characteristic that enables individuals to view themselves without judgment (Cong and Gao, 1999). Previous research has linked SA to lower emotional distress and better mental health outcomes (Su et al., 2025), and emerging evidence suggests that SA may be compromised by excessive social comparison on social media (Tseng and Huang, 2025).

According to self-determination theory (SDT) (Deci and Ryan, 2012), when the basic needs for autonomy, competence, and relatedness are satisfied, individuals show better psychological wellbeing and social adjustment. Need satisfaction tends to accompany stronger intrinsic motivation and greater psychological resilience. In contrast, need frustration is often associated with psychological distress and maladjustment. Within this framework, problematic social network use (PSNU) can be understood as a non-self-determined behavioral pattern that displaces genuine social interactions with shallow online activities. Such patterns of use are likely to compromise rather than fulfill basic psychological needs, particularly the need for relatedness. PNS directly captures the degree to which these three basic needs are fulfilled. Within SDT, the organismic integration process occupies a core position, where the fulfillment of fundamental psychological needs is closely tied to supplying the necessary psychological resources for cultivating a unified, cohesive, and enduring self-perception. This process involves the gradual internalization of values and the formation of a unified self-structure, which is a prerequisite for optimal functioning and wellbeing. Self-acceptance, in this view, represents the successful outcome of this integrative process. From this perspective, PNS is not merely about feeling good; it is about furnishing the internal resources necessary for healthy self-development. Importantly, this theoretical account suggests that PNS serves as a foundational precursor to self-acceptance (SA). When psychological needs are consistently met, individuals are more likely to assimilate experiences into a congruent self-concept, leading to a non-judgmental and accepting stance toward themselves. Conversely, when needs are frustrated, the self becomes fragmented, and individuals may develop harsh self-criticisms and a diminished capacity for self-acceptance. LON, from this SDT perspective, can be understood as an affective consequence of relatedness need frustration. Marttila (2024) provided further support for this reasoning by identifying unmet social needs as a central motivation underlying PSNU and by suggesting a dynamic, mutually reinforcing association between PSNU and LON.

Recent evidence has further expanded this line of inquiry by identifying multiple psychological pathways linking PSNU to LON and related mental health outcomes. During the COVID period, Çiçek et al. (2024) found social support to be a mediator between excessive social media use and depressive symptoms, with relational resource levels also covarying inversely with PSNU-related adverse outcomes. Similarly, Çiçek (2021) demonstrated that self-esteem mediated the association between LON and psychological wellbeing among university students, suggesting that positive self-evaluation functions as a protective psychological resource. Extending these findings, Ünsal et al. (2025) reported that self-esteem and positive childhood experiences sequentially mediated the relationship between PSNU and LON, highlighting the interplay between developmental factors and self-perceptions. Likewise, Çiçek et al. (2026) identified meaning in life and resilience as sequential mediators linking LON to PSNU, indicating that existential meaning and coping resources may also play protective roles. Taken together, these recent investigations converge on a common theme: the association between PSNU and LON operates through multiple, sequentially organized psychological mechanisms spanning self-evaluation, existential meaning, and psychological resilience. However, despite this growing body of evidence, the specific sequential pathway through basic psychological need satisfaction followed by self-acceptance, a pathway directly grounded in SDT’s organismic integration process, has not yet been systematically examined.

Drawing on both the SDT framework and the empirical evidence reviewed above, PSNU may first be associated with lower PNS. This sense of psychological deprivation may, in turn, be related to lower levels of SA. Reduced SA may then leave individuals with fewer emotional regulation and coping resources when they encounter social difficulties, which may be associated with stronger feelings of LON. Accordingly, this study proposes that PNS and SA may form a serial mediation pathway between PSNU and LON among college students.

Critically, previous studies have separately examined the direct association between PSNU and LON (Fioravanti et al., 2025; Wang et al., 2025), the negative association between PSNU and PNS (Tseng and Huang, 2025; Zhang et al., 2024), and the positive role of SA in mental health (Morales-Rodríguez et al., 2020; Xu et al., 2025). However, these lines of research have largely remained independent, leaving the integrated, sequential mechanism through which PSNU relates to LON via both motivational and self-evaluative pathways unexplored. The present study addresses this gap by proposing and testing a serial mediation model in which PNS and SA function as sequential mediators linking PSNU to LON. This integrated framework not only extends prior work that has focused on direct effects or single mediators, but also identifies potential targets for mental health interventions in college settings. Specifically, interventions designed to enhance students’ PNS and SA may help disrupt the pathway from PSNU to LON at different stages of the cascading process.

**FIGURE 1:**
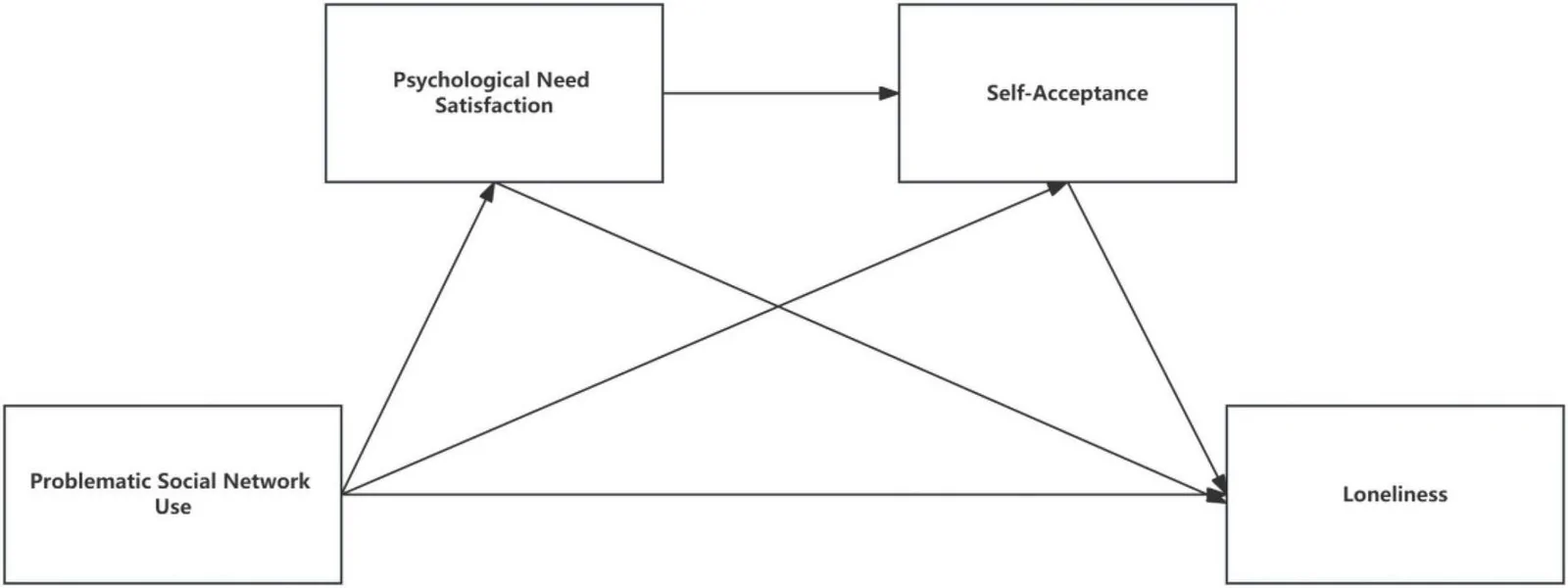
Proposed conceptual framework of the present study.

Taken together, this theoretical integration suggests that PSNU may relate to LON through three distinct pathways: a motivational pathway through PNS, a self-evaluative pathway through SA, and a sequential pathway through both. We therefore propose the following hypotheses (see Figure 1):

*H1*: PSNU is significantly and positively associated with LON.

*H2*: PNS mediates the association between PSNU and LON.

*H3*: SA mediates the association between PSNU and LON.

*H4*: PNS and SA play a serial mediating role in the association between PSNU and LON.

## 2 Materials and methods

### 2.1 Participants and data collection procedure

From November to December 2025, participants were enrolled via an online survey hosted on the Wenjuanxing platform^1^. Data were collected from students enrolled at three public universities located in Zhejiang, a region in Southeast China. Given the geographic and cultural diversity within China, the present sample is best understood as representing university students in this specific regional context, and generalizations to other Chinese populations should be made with caution. Initial contact was established with university administrators, followed by coordination with academic counselors. Counselors introduced the purpose of the survey during class meetings and invited students to participate on a voluntary basis. Classes were selected from available rosters via a computerized procedure, yet survey participation depended on students’ voluntary completion of the online questionnaire, with the link shared through class WeChat groups. Written informed consent was obtained from all participants aged 18 and above prior to survey completion. For participants under 18 years of age (10.6% of the sample), written informed consent was obtained from their parents or legal guardians, and student assent was also obtained prior to participation. The study protocol was approved by the Institutional Review Board and conducted in accordance with the Declaration of Helsinki. All respondents were notified that their submissions were anonymous and kept confidential, with no incentives involved.

According to Kline (2018), an approximate guideline of 10 respondents per questionnaire item was used to inform sample size planning. The survey included 79 items, requiring approximately 790 valid responses. Accounting for an anticipated 20% invalid response rate (e.g., missing data or uniform response patterns), we targeted approximately 948 participants [790 + (790 × 20%) = 948]. The survey yielded 1,100 completed questionnaires in total. After data screening, 108 responses were excluded due to missing data or uniform response patterns, resulting in 992 valid questionnaires for analysis (valid response rate = 90.18%).

The obtained sample (*N* = 992) exceeded the minimum requirement of 790 valid responses estimated by the 10:1 ratio guideline (Kline, 2018), providing sufficient statistical power for the proposed mediation analyses. The sample comprised university students from diverse grade levels (freshmen to seniors) and academic majors (humanities, social sciences, and natural sciences), which was deemed appropriate for examining the hypothesized relationships among PSNU, PNS, SA, and LON in the target population. Preliminary analyses confirmed that the data met key statistical assumptions, including normality and absence of multicollinearity, supporting the appropriateness of the planned parametric analyses (PROCESS Model 6 and CFA). Table 1 presents the final sample of 992 university students, comprising 504 women (50.8%) and 488 men (49.2%) and a mean age of 20.31 years (SD = 1.48). As age was collected using categorical response options (“Below 18,” “18–20,” “Above 20”), the mean age and standard deviation were estimated using group midpoints (17.5, 19.0, and 21.5 years, respectively).

**TABLE 1:** Demographic characteristics of the participants.

Variable	*N*	Percentage (%)
Gender		
Male	488	49.2%
Female	504	50.8%
Age		
Below 18	105	10.6%
18–20	305	30.7%
Above 20	582	58.7%
Grade level		
Freshman	201	20.3%
Sophomore	372	37.5%
Junior	256	25.8%
Senior	163	16.4%
Major		
Humanities	305	30.7%
Social sciences	317	32.0%
Natural sciences	370	37.3%

### 2.2 Measures

#### 2.2.1 Problematic social network use

This study used the Problematic Mobile Social Network Use Questionnaire (PMSNUQ) developed by Jiang (2018), which aims to evaluate the potential adverse effects associated with individuals’ engagement in mobile-based social networking. The 20-item scale adopts a five-point Likert format, with higher scores reflecting greater levels of problematic use. Sample items include “I find it difficult to control my time spent on social networking sites” and “I feel anxious when I cannot access social networking sites.” The instrument’s validity and reliability were supported by earlier studies using Chinese respondents (Ding et al., 2024). The Cronbach’s alpha for the PMSNUQ was 0.952.

#### 2.2.2 Psychological need satisfaction

PNS was assessed using the Basic Psychological Needs Scale (BPNS) developed by Liu et al. (2013), which measures the extent to which individuals perceive their basic psychological needs. The scale includes 19 items rated on a 7-point Likert scale, ranging from 1 (not at all true) to 7 (completely true), with higher scores indicating greater satisfaction. Sample items include “I feel free to express my own opinions” (autonomy), “I am good at the things I do” (competence), and “I feel connected to people who care about me” (relatedness). Previous work has demonstrated the instrument’s validity and reliability within Chinese populations (Xiao and Zheng, 2022). The Cronbach’s alpha for the BPNS was 0.956.

#### 2.2.3 Self-acceptance

SA was measured using the Self-Acceptance Questionnaire (SAQ) developed by Cong and Gao (1999), a 16-item self-report instrument widely applied in psychological, educational, and sociological research. Responses were recorded using a 4-point Likert scale, where higher ratings signified greater SA. The scale comprises two subdimensions: the Self-Evaluation Factor, which captures individuals’ positive self-appraisals (positively scored), and the SA Factor, which assesses acceptance of personal shortcomings (reverse-scored). Sample items include “Overall, I am satisfied with myself” (positively scored) and “I often worry that others will look down on me” (reverse-scored). Earlier investigations have affirmed the instrument’s sound validity and reliability among Chinese groups (Wu et al., 2024). In the current study, the Cronbach’s alpha for the SAQ was 0.937.

#### 2.2.4 Loneliness

LON was measured using the UCLA Loneliness Scale developed by Russell et al. (1980), a widely adopted instrument with established reliability and validity across diverse cultural contexts. This 20-item instrument uses a 4-point Likert format (1–4), with nine items reverse-keyed to minimize response set effects. Sample items include “How often do you feel that you lack companionship?” and “How often do you feel isolated from others?” Higher total scores reflect greater perceived LON. The scale’s validity and reliability have received prior support within Chinese samples (Fang et al., 2023). In this study, the Cronbach’s alpha for the UCLA Loneliness Scale was 0.951.

## 3 Data analysis

The dataset was examined using IBM SPSS 26.0 and AMOS 26.0 across several analytical stages. First, a check for common method variance was carried out through Harman’s one-factor test. Second, Pearson correlation analysis assessed associations among PSNU, PNS, SA, and LON. Third, the measurement model was evaluated using CFA conducted with AMOS. Gender, age, grade level, and major were included as covariates. Using PROCESS Model 6, we examined whether PNS and SA sequentially mediated the link from PSNU to LON. Indirect effects were assessed with 5,000 bootstrap resamples and 95% bias-corrected CIs (Preacher and Hayes, 2008), with significance indicated when the interval excluded zero (Hayes and Rockwood, 2017).

## 4 Results

### 4.1 Common method bias

Given that all measures relied on self-reports, CMB was a potential concern. Harman’s single-factor test served as an initial diagnostic check. The analysis yielded five factors having eigenvalues exceeding 1.0, and the largest factor captured 32.632% of the total variance, falling under the conventional 40% criterion. These results suggest that CMB may not substantially influence the observed associations in the present study (Podsakoff and Organ, 1986).

An Unmeasured Latent Method Construct (ULMC) test in AMOS 26.0 showed that adding a common method factor to the four-factor model (χ^2^/df = 1.411, RMSEA = 0.020, CFI = 0.975) produced minimal fit changes (ΔCFI = 0.006, ΔRMSEA = 0.002). The ULMC model still fit well (χ^2^/df = 1.321, RMSEA = 0.018, CFI = 0.981), within recommended limits (Abd-El-Fattah, 2010). Thus, CMB does not appear to be a major concern.

### 4.2 Multicollinearity test

As shown in Table 2, tolerance statistics all exceeded 0.10, and VIF values all remained under 3.30, pointing to an absence of significant multicollinearity issues in the current data (Hair et al., 2019).

**TABLE 2:** Multicollinearity diagnostics of the structural model.

Constructs	Tolerance	VIF
PSNU	0.776	1.288
PNS	0.672	1.487
SA	0.755	1.324

PSNU, Problematic Social Network Use; PNS, Psychological Need Satisfaction; SA, Self-Acceptance. VIF, Variance Inflation Factor.

### 4.3 Confirmatory factor analysis

To evaluate the measurement structure, we conducted CFA to check whether observed items loaded onto their respective latent constructs as expected. The four-factor measurement structure yielded satisfactory fit indices: χ^2^/df = 1.411, RMSEA = 0.020, GFI = 0.912, AGFI = 0.902, CFI = 0.975, IFI = 0.973, TLI = 0.975. All indices were within acceptable ranges (Hu and Bentler, 1999), supporting the model’s representation of the theoretical constructs.

### 4.4 Composite reliability and validity

As shown in Table 3, all item factor loadings for the four scales (PSNU, PNS, SA, and LON) exceed 0.7, and the Cronbach’s α (ranging from 0.937 to 0.956), composite reliability (CR, ranging from 0.944 to 0.960), and AVE values (ranging from 0.515 to 0.558) all meet the recommended thresholds, indicating that all four scales possess good reliability and convergent validity (Krzanowski, 2000). As summarized in Table 4, the Heterotrait-Monotrait (HTMT) ratios for all construct pairs ranged from 0.350 to 0.597, remaining beneath the 0.85 benchmark and confirming adequate discriminant validity (Henseler et al., 2015). As additionally displayed in Table 5, each construct’s AVE square root ranged from 0.718 to 0.747, exceeding the corresponding correlations with other constructs, which further supports discriminant validity across the four scales (Fornell and Larcker, 1981).

**TABLE 3:** Reliability and validity.

Constructs	Items	Standardized loadings	CR	Cronbach’s α	AVE
PSNU	PSNU 1	0.735	0.956	0.952	0.522
	PSNU 2	0.727			
	PSNU 3	0.724			
	PSNU 4	0.728			
	PSNU 5	0.727			
	PSNU 6	0.705			
	PSNU 7	0.710			
	PSNU 8	0.726			
	PSNU 9	0.743			
	PSNU 10	0.729			
	PSNU 11	0.703			
	PSNU 12	0.707			
	PSNU 13	0.721			
	PSNU 14	0.712			
	PSNU 15	0.734			
	PSNU 16	0.715			
	PSNU 17	0.736			
	PSNU 18	0.720			
	PSNU 19	0.708			
	PSNU 20	0.743			
PNS	PNS 1	0.733	0.960	0.956	0.558
	PNS 2	0.718			
	PNS 3	0.713			
	PNS 4	0.761			
	PNS 5	0.767			
	PNS 6	0.750			
	PNS 7	0.751			
	PNS 8	0.723			
	PNS 9	0.764			
	PNS 10	0.731			
	PNS 11	0.778			
	PNS 12	0.760			
	PNS 13	0.748			
	PNS 14	0.730			
	PNS 15	0.772			
	PNS 16	0.761			
	PNS 17	0.750			
	PNS 18	0.725			
	PNS 19	0.754			
SA	SA 1	0.702	0.944	0.937	0.515
	SA 2	0.732			
	SA 3	0.712			
	SA 4	0.728			
	SA 5	0.715			
	SA 6	0.719			
	SA 7	0.714			
	SA 8	0.706			
	SA 9	0.701			
	SA 10	0.712			
	SA 11	0.715			
	SA 12	0.709			
	SA 13	0.736			
	SA 14	0.738			
	SA 15	0.711			
	SA 16	0.730			
LON	LON 1	0.712	0.955	0.951	0.516
	LON 2	0.717			
	LON 3	0.712			
	LON 4	0.705			
	LON 5	0.711			
	LON 6	0.716			
	LON 7	0.721			
	LON 8	0.729			
	LON 9	0.720			
	LON 10	0.740			
	LON 11	0.736			
	LON 12	0.716			
	LON 13	0.713			
	LON 14	0.725			
	LON 15	0.711			
	LON 16	0.704			
	LON 17	0.733			
	LON 18	0.702			
	LON 19	0.709			
	LON 20	0.730			

N, 992. PSNU, Problematic Social Network Use; PNS, Psychological Need Satisfaction; SA, Self-Acceptance; LON, Loneliness; CR, Composite reliability; AVE, Average variance extracted. The same applies to the following tables.

**TABLE 4:** HTMT.

Constructs	LON	PNS	PSNU	SA
LON				
PNS	0.597			
PSNU	0.533	0.477		
SA	0.582	0.506	0.350	

**TABLE 5:** Fornell-Larcker criterion.

Constructs	LON	PNS	PSNU	SA
LON	***0.718***			
PNS	−0.571	***0.747***		
PSNU	0.508	−0.456	***0.723***	
SA	−0.551	0.480	−0.332	***0.718***

The bold and italicized diagonal values represent the square root of the average variance extracted for each construct.

### 4.5 Correlations among study variables

Table 6 presents the means, SDs, skewness, kurtosis, and intercorrelations for all four variables. All skewness and kurtosis values fell within acceptable limits (| skewness| < 2, | kurtosis| < 7), suggesting roughly normal distributions (Curran et al., 1996). All correlations were significant.

**TABLE 6:** Descriptive overview and variable intercorrelations.

Variable	*M*	*SD*	SK	Kur	1	2	3	4
1 PSNU	2.965	0.670	0.104	−0.072	1	1	1	1
2 PNS	4.058	1.013	−0.015	0.048	−0.455***			
3 SA	2.532	0.544	−0.142	−0.155	−0.331***	0.478***		
4 LON	2.516	0.539	−0.233	−0.060	0.507***	−0.569***	−0.550***	

***p < 0.001; same below.

### 4.6 Mediation effect analysis

All regression analyses adjusted for gender, age, grade, and major as covariates. Table 7 presents the regression outcomes for the mediation analysis. PSNU was significantly negatively associated with PNS (β = −0.454, *p* < 0.001, *R*^2^ = 0.213). For SA, both PSNU (β = −0.141, *p* < 0.001) and PNS (β = 0.415, *p* < 0.001) were significant correlates (*R*^2^ = 0.250). For LON, PSNU (β = 0.267), PNS (β = −0.293), and SA (β = −0.321) all showed significant associations with LON (all *p* < 0.001, *R*^2^ = 0.481). All bootstrap CIs excluded zero. These findings support H1.

**TABLE 7:** Regression analysis of mediator variables.

Result variable	Independent variable	β	SE	*T*	Bootstrap 95% CI		*P*	*R* ^2^	*F*
					LLCI	ULCI			
PNS	PSNU	−0.454	0.028	−16.074	−0.510	−0.399	< 0.001	0.213	53.322
SA	PSNU	−0.141	0.031	−4.536	−0.202	−0.080	< 0.001	0.250	54.816
	PNS	0.415	0.031	13.357	0.354	0.476	<0.001		
LON	PSNU	0.267	0.026	10.239	0.216	0.318	< 0.001	0.481	130.301
	PNS	−0.293	0.028	−10.406	−0.348	−0.238	<0.001		
	SA	−0.321	0.027	−12.119	−0.374	−0.269	<0.001		

*N*, 992. β, Standardized regression coefficient; SE, Standard Error; *t*, t-statistic; CI, Confidence Interval; LLCI, Lower Limit of Confidence Interval; ULCI, Upper Limit of Confidence Interval; *R*^2^, Coefficient of Determination; *F*, F-statistic. Bootstrap confidence intervals were based on 5,000 bootstrap samples. All *p*-values are two-tailed.

**FIGURE 2:**
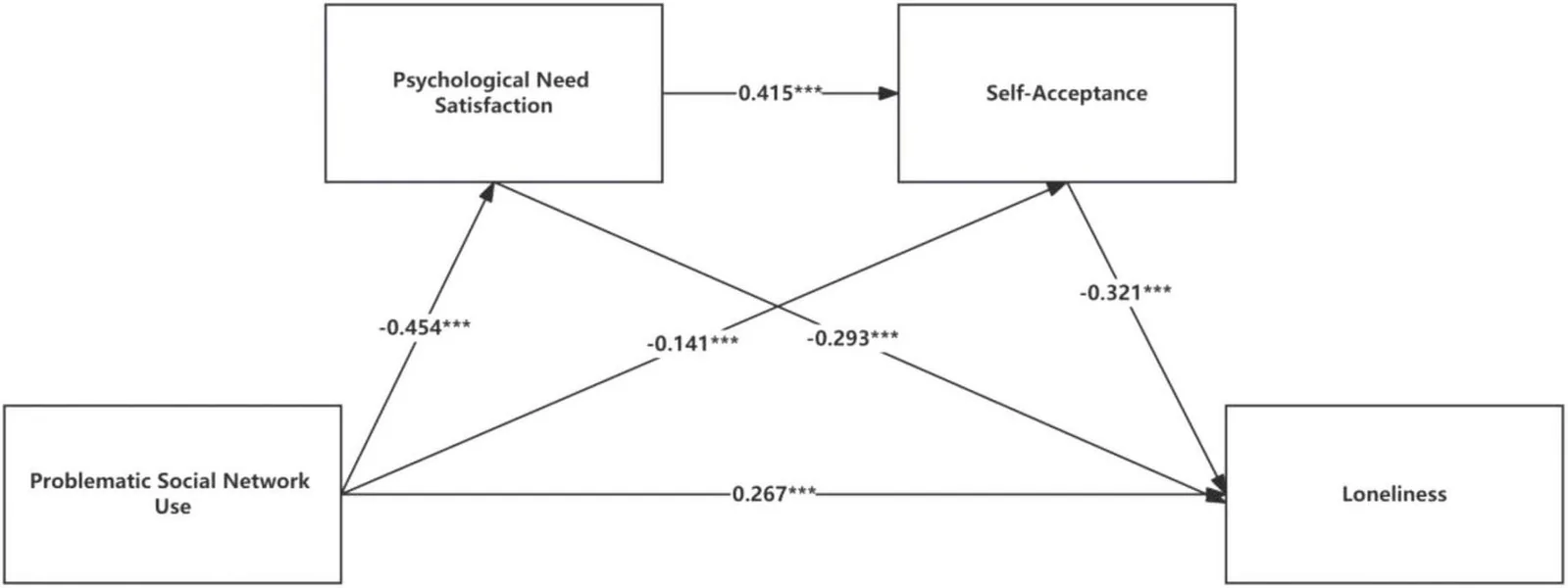
Mediation model. ***p < 0.001.

Table 8 displays the total, direct, and indirect effects linking PSNU to LON. The total effect was significant (β = 0.506, *95% CI* [0.452, 0.560]), and the direct effect also reached significance (β = 0.267, *95% CI* [0.216, 0.318]), representing 52.77% of the total. The indirect associations through PNS (β = 0.133), through SA (β = 0.045), and the serial mediation through PNS and SA (β = 0.061) were all significant, accounting for 26.29, 8.89, and 12.06% of the total association, respectively (see Figure 2 for the path model). All bootstrap CIs excluded zero. Thus, H2, H3, and H4 are supported.

**TABLE 8:** Serial mediation of psychological need satisfaction and self-acceptance between problematic social network use and loneliness.

Effect	Path	β	SE	Bootstrap 95% CI		Percentage (%)
				LLCI	ULCI	
Total effect	PSNU → LON	0.506	0.027	0.452	0.560	100.00
Direct effect	PSNU → LON	0.267	0.026	0.216	0.318	52.77
Indirect effect	PSNU → PNS → LON	0.133	0.016	0.104	0.165	26.28
	PSNU → SA → LON	0.045	0.011	0.024	0.067	8.89
	PSNU → PNS → SA → LON	0.061	0.008	0.046	0.078	12.06

Among the indirect paths, the route via PNS alone (26.28%) contributed the most to the total association, followed by the serial route through both PNS and SA (12.06%), and the single SA pathway (8.89%). These results point to PNS as the dominant mediating pathway, with SA playing a secondary yet still significant role in the association under investigation. Importantly, the sequential indirect effect (12.06%) was larger than the standalone effect of SA, underscoring the value of examining the cascading process rather than focusing on each mediator in isolation.

## 5 Discussion

The present findings indicated that PSNU was positively associated with LON among university students, consistent with H1. This result aligns with O’Brien et al. (2023), who reported that greater engagement in PSNU is linked to elevated levels of LON. Within the context of SDT, this association may reflect how university students experience and interpret social connectedness in relation to their online social engagement, rather than the overall frequency of social interaction (Deci and Ryan, 2012). Specifically, higher levels of PSNU among university students may be associated with perceptions of social interaction that are experienced as less meaningful or less satisfying, which co-occur with higher levels of LON (Zhou and Shen, 2024). This finding is also consistent with recent work by Çiçek et al. (2024), who found that problematic social media use was significantly associated with depressive symptoms, a correlate often closely linked with LON. Collectively, these convergent findings across different cultural contexts and samples suggest that the positive association between PSNU and LON is robust and warrants attention in both research and intervention efforts.

The present findings indicated that PNS was statistically involved in the association between PSNU and LON, which is consistent with H2. In line with Tseng and Huang (2025) and Huang et al. (2024), higher PSNU scores corresponded to lower PNS scores, and reduced PNS levels were linked to elevated LON scores. From a SDT perspective, this pattern may be understood in terms of how problematic forms of social network use are related to individuals’ experiences of autonomy, competence, and relatedness (Deci and Ryan, 2012). SDT emphasizes that meeting these core needs (autonomy, competence, and relatedness) is closely tied to social bonding and overall psychological wellness. When PSNU is characterized by avoidant or compensatory tendencies, it may be less closely aligned with experiences of psychological need satisfaction, particularly with respect to relatedness and competence (Ostendorf et al., 2020; Parent, 2023). Under these conditions, online encounters may feel less emotionally fulfilling, a feature that relates to elevated LON (Sun et al., 2023). The mediating function of PNS observed in this study complements the earlier findings of Çiçek et al. (2026), which identified resilience and meaning in life as additional mediators within the PSNU-LON association. While their study focused on positive psychological resources that buffer against LON, our findings emphasize the motivational deficits, specifically the frustration of basic needs, that may underlie this association. This suggests that both the presence of psychological resources and the absence of need frustration are important pathways linking PSNU to LON. The indirect association through PNS accounted for 26.28% of the total association. This effect size positions need satisfaction as a key component within the online social behavior-emotional wellbeing relationship, and as a possible focus for intervention efforts. Collectively, the current evidence points to PNS as a pertinent correlate within the linkage from PSNU to LON in college populations, emphasizing that psychological need satisfaction deserves attention in research on this topic.

Evidence also corroborated the mediating function of SA between PSNU and LON, thereby supporting H3. This finding aligns with earlier work showing SA as a buffer against adverse emotional states (Morales-Rodríguez et al., 2020; Su et al., 2025). This investigation extends prior research by embedding SA within the PSNU context, with findings indicating that PSNU is linked to lower SA levels, a connection paralleled by increased social comparison. With continuous exposure to idealized portrayals of others’ lives, students tend to gauge themselves against unrealistic benchmarks, and reduced SA is commonly observed alongside such self-appraisals (Tseng and Huang, 2025). This account aligns with SDT’s organismic integration process. Need satisfaction corresponds to favorable self-evaluations, whereas need frustration is linked to more frequent negative self-appraisals (Zhang et al., 2024). The role of SA as a mediator is further supported by research showing that self-esteem, a closely related self-evaluative construct, plays a similar mediating role between LON and wellbeing (Çiçek, 2021) and between PSNU and LON (Ünsal et al., 2025). These convergent findings suggest that self-evaluative processes are consistently implicated in the relationship between PSNU and emotional outcomes, regardless of whether they are operationalized as self-esteem or self-acceptance. In the Chinese cultural context, where self-criticism is often encouraged as a means of self-improvement, the erosion of SA may be particularly consequential, as students may lack culturally endorsed strategies for maintaining positive self-regard in the face of social comparison. Moreover, the emphasis on social harmony and group acceptance in Chinese culture may amplify the negative consequences of diminished self-acceptance, as students may experience additional distress from perceived failure to meet social expectations (Fan et al., 2024). This finding points to the usefulness of enhancing SA as a psychological resource in intervention settings, particularly within cultural contexts where self-criticism is prevalent.

The present study identified a serial mediation pathway through PNS followed by SA, supporting H4. This sequential mechanism suggests that the association between PSNU and LON operates through a cascading process in which motivational deficits precipitate self-evaluative deficits. The theoretical logic underlying this pathway is that PNS provides the psychological foundation for SA. In the absence of sufficient autonomy, competence, and relatedness need fulfillment, students may lack the internal foundation required for nonjudgmental self-acceptance (Deci and Ryan, 2012). This account aligns with prior evidence that PSNU replaces genuine social contact with shallow online activities, a pattern that corresponds with diminished PNS (Tseng and Huang, 2025), which in turn deprives individuals of the psychological resources needed for positive self-regard (Morales-Rodríguez et al., 2020). The decline in SA further compromises students’ ability to cope with social challenges, ultimately contributing to LON (Li et al., 2021). This serial mediation model advances theoretical understanding by integrating motivational and self-evaluative processes within a single framework. Prior studies have typically examined these mechanisms in isolation; the present study demonstrates that they operate sequentially, offering a more nuanced explanation of how PSNU relates to LON. The sequential indirect path accounted for 12.06% of the total effect, exceeding the single SA path at 8.89%. This underscores the value of examining the cascading process rather than focusing on each mediator in isolation. This suggests that interventions targeting both PNS and SA sequentially may be more effective than addressing either in isolation. For instance, interventions that first help students restore basic need satisfaction, for example through fostering meaningful social connections, may create the psychological conditions necessary for enhancing self-acceptance, which in turn may reduce LON more effectively than a single-focus approach. In the Chinese cultural setting, social evaluation carries considerable weight, making this cascading process potentially more evident. When students experience need frustration, they may encounter internal resource shortages alongside external demands to uphold social status and avoid face loss in interpersonal encounters. The identification of this sequential pathway also provides a potential explanation for why some individuals are more vulnerable to the negative effects of PSNU than others: those with lower baseline levels of need satisfaction may be particularly susceptible to the cascading effects described in our model. Future research could explore individual differences, such as personality traits or attachment styles, that may moderate this sequential process.

## 6 Implications and limitations

### 6.1 Theoretical implications

First, this study extends Self-Determination Theory to the context of PSNU among university students by demonstrating how online social behavior is associated with PNS and LON during emerging adulthood. By focusing on university students, the present findings broaden SDT-based accounts of psychological need processes beyond traditional offline settings, highlighting the relevance of digital social environments for understanding LON in early adulthood. Moreover, the serial mediation structure observed here indicates that motivational deficiencies may correspond with self-evaluative deficiencies, which are then linked to elevated LON. This integrated framework extends prior work that largely examined these mechanisms separately. It offers a fuller picture of how external social behaviors and internal self-evaluative processes jointly relate to emotional outcomes within the SDT framework.

Second, this study advances SDT by identifying SA as a sequential mediator in the association between PSNU and LON. Within the SDT framework, the organismic integration process posits that basic psychological need satisfaction provides the foundation for individuals to develop coherent and non-judgmental self-evaluation. These results empirically back this theoretical account, positioning PNS as a necessary precursor to SA. When PSNU impairs need satisfaction, individuals experience psychological deprivation that compromises their capacity for SA, which in turn contributes to LON. By specifying the sequential link from need satisfaction to SA in digital environments, this serial mediation model offers a fresh contribution to SDT. This particular aspect has received limited empirical investigation thus far.

Third, this work adds to the broader PSNU literature by identifying the distinct yet interrelated psychological pathways from PSNU to LON, all rooted in SDT. Whereas much of the existing research has focused on direct associations or broad correlational patterns, the present study identifies three distinct pathways within the SDT framework: a motivational pathway through PNS, a self-evaluative pathway through SA, and a sequential pathway through both mediators. This differentiated understanding may help explain why some individuals are more vulnerable to LON in the context of PSNU than others. By situating this model within the Chinese cultural context, the present study also provides preliminary evidence that cultural factors, particularly the emphasis on social harmony and group acceptance, may amplify the negative consequences of PSNU, highlighting the importance of cultural sensitivity in both theory development and intervention design within SDT-based research.

### 6.2 Practical implications

At the individual level, these results point to the value of helping students recognize and regulate their PSNU, especially behaviors tied to emotional coping and social comparison. Encouraging students to engage in offline social activities that foster meaningful social connections should be the primary focus, as this directly addresses the need satisfaction pathway that accounts for the largest indirect effect. For students who already experience compromised SA, supplementary interventions such as cognitive-behavioral techniques or self-compassion training may provide additional benefit, although the relatively smaller standalone effect of SA suggests that these should complement, rather than substitute for, need-based interventions.

At the university level, institutions should consider integrating interventions that promote psychological wellbeing by focusing on PNS and SA. Providing workshops or counseling services aimed at enhancing students’ SA, as well as offering programs that facilitate face-to-face social interactions, could help students build stronger social networks and improve emotional stability. Importantly, the serial nature of the mediating pathway suggests that multi-component interventions addressing both motivational and self-evaluative processes may yield greater benefits than single-focus approaches, as they can interrupt the cascading process at multiple points. Mental health initiatives focused on social media use, such as digital wellness courses, may further assist students in regulating their online engagement.

At the societal level, there is a need for broader initiatives to improve media literacy and promote responsible social media use. Public campaigns and educational programs that raise awareness about the potential psychological risks of excessive social network use, as well as the importance of SA, could encourage healthier online behaviors. Partnerships between universities, mental health organizations, and social media platforms could help create a more supportive environment for students, reducing LON and promoting overall psychological wellbeing. Given the high prevalence of social media use among young adults, such cooperative approaches may prove useful at the population level, with potential relevance for reducing mental health disparities tied to PSNU.

### 6.3 Limitations and future directions

Several limitations warrant mention. First, the cross-sectional nature of this study does not allow for causal conclusions about the relationships among PSNU, PNS, SA, and LON. Although our serial mediation model was grounded in a robust theoretical framework (SDT), the observed statistical pathways could potentially be bidirectional. For example, LON may also predict increased PSNU as individuals seek social connection online (Çiçek et al., 2026). Future work using longitudinal or experimental designs is required to establish temporal precedence and causal direction. Second, self-report measures carry the risk of social desirability and recall inaccuracies. While common method bias was examined and deemed unlikely to be a major issue, subsequent studies might include other-report assessments (e.g., peer evaluations of SA, parent observations of social functioning) or objective usage data (e.g., screen time records) to diversify data sources and strengthen the testing of the proposed model. Triangulating data from multiple sources would provide a more robust test of the hypothesized relationships. Third, participants were drawn from three universities in Zhejiang, a province in Southeast China. This restricts the extent to which the current findings can be generalized to other parts of the country or to different cultural settings. Given China’s substantial regional diversity in economic development, educational resources, and cultural norms (Fan et al., 2024), the unique characteristics of the Zhejiang region may not be representative of other Chinese provinces. Cross-regional comparisons within China (e.g., comparing eastern vs. western provinces) and cross-cultural replications (e.g., in individualistic vs. collectivistic cultures) are necessary to establish the generalizability of the present findings. Fourth, factors like self-esteem, social support, and emotion regulation represent other potentially relevant psychological dimensions that deserve future attention. Subsequent investigations may incorporate these variables into broader analytical models to explore their joint functioning with PNS and SA in relation to LON among university populations. Finally, future research could develop and evaluate intervention strategies targeting PNS and SA to mitigate the negative consequences of PSNU, providing evidence-based solutions to reduce LON among university students. Specifically, randomized controlled trials could compare the effectiveness of need-focused interventions (e.g., promoting meaningful social connections) versus self-acceptance interventions (e.g., self-compassion training), as well as their sequential combination, to identify the most effective approach for reducing LON in this population.

## 7 Conclusion

Guided by Self-Determination Theory, the present work examined the association from PSNU to LON in a Zhejiang -based university student sample, with emphasis on the serial mediating functions of PNS and SA. The findings revealed that PSNU was positively associated with LON through three distinct pathways: independently through PNS (26.28%), independently through SA (8.89%), and sequentially through both mediators (12.06%). These results suggest that PNS serves as the primary mechanism linking PSNU to LON, while SA plays a complementary role as a downstream consequence of need deprivation. Theoretically, these findings extend SDT to the digital context by demonstrating that online social behaviors are linked to emotional wellbeing through both motivational and self-evaluative pathways, providing empirical support for SDT’s organismic integration process. Practically, interventions targeting LON among university students may benefit from a sequential approach that first addresses PNS and subsequently fosters SA. Although limited by a cross-sectional design and self-report measures, this work still provides theoretical and applied insights for mental health promotion within higher education settings. Subsequent work could adopt longitudinal approaches and cross-cultural contrasts to provide additional support for the present observations.

## Data Availability

The raw data supporting the conclusions of this article will be made available by the authors, without undue reservation.

## Footnotes

1
https://www.wjx.cn/
